# Nicotine patch preloading for smoking cessation (the preloading trial): study protocol for a randomized controlled trial

**DOI:** 10.1186/1745-6215-15-296

**Published:** 2014-07-22

**Authors:** Nicola Lindson-Hawley, Tim Coleman, Graeme Docherty, Peter Hajek, Sarah Lewis, Deborah Lycett, Andy McEwen, Hayden McRobbie, Marcus R Munafò, Steve Parrott, Paul Aveyard

**Affiliations:** 1 Nuffield Department of Primary Care Health Sciences, University of Oxford, Radcliffe Observatory Quarter, Woodstock Road, OX2 6GG Oxford, UK; 2 Division of Primary Care, University of Nottingham, Queen’s Medical Centre, NG7 2UH Nottingham, UK; 3 Division of Epidemiology & Public Health, University of Nottingham, Clinical Sciences Building, Nottingham City Hospital, Hucknall Road, NG5 1PB Nottingham, UK; 4 Tobacco Dependence Research Unit, Wolfson Institute of Preventive Medicine, Barts and The London School of Medicine and Dentistry, 55 Philpot Street, Whitechapel, E1 2HJ London, UK; 5 Faculty of Health and Life Sciences, Richard Crossman Building, Coventry University, Priory Street, CV1 5FB Coventry, UK; 6 Health Behaviour Research Centre, Department of Epidemiology & Public Health, UCL, Gower Street, WC1E 6BT London, UK; 7 School of Experimental Psychology, University of Bristol, 12a Priory Road, BS8 1TU Bristol, UK; 8 MRC Integrative Epidemiology Unit (IEU), University of Bristol, 12a Priory Road, BS8 1TU Bristol, UK; 9 Department of Health Sciences, University of York, Seebohm Rowntree Building, Heslington, YO10 5DD York, UK

**Keywords:** Smoking, Nicotine, Preloading, Tobacco, Cessation, Quitting, Addiction, Treatment

## Abstract

**Background:**

The use of nicotine replacement therapy before quitting smoking is called nicotine preloading. Standard smoking cessation protocols suggest commencing nicotine replacement therapy only on the first day of quitting smoking (quit day) aiming to reduce withdrawal symptoms and craving. However, other, more successful smoking cessation pharmacotherapies are used prior to the quit day as well as after. Nicotine preloading could improve quit rates by reducing satisfaction from smoking prior to quitting and breaking the association between smoking and reward. A systematic literature review suggests that evidence for the effectiveness of preloading is inconclusive and further trials are needed.

**Methods/Design:**

This is a study protocol for a multicenter, non-blinded, randomized controlled trial based in the United Kingdom, enrolling 1786 smokers who want to quit, funded by the National Institute for Health Research, Health Technology Assessment program, and sponsored by the University of Oxford. Participants will primarily be recruited through general practices and smoking cessation clinics, and randomized (1:1) either to use 21 mg nicotine patches, or not, for four weeks before quitting, whilst smoking as normal. All participants will be referred to receive standard smoking cessation service support.

Follow-ups will take place at one week, four weeks, six months and 12 months after quit day. The primary outcome will be prolonged, biochemically verified six-month abstinence. Additional outcomes will include point prevalence abstinence and abstinence of four-week and 12-month duration, side effects, costs of treatment, and markers of potential mediators and moderators of the preloading effect.

**Discussion:**

This large trial will add substantially to evidence on the effectiveness of nicotine preloading, but also on its cost effectiveness and potential mediators, which have not been investigated in detail previously. A range of recruitment strategies have been considered to try and compensate for any challenges encountered in recruiting the large sample, and the multicentre design means that knowledge can be shared between recruitment teams. The pragmatic study design means that results will give a realistic estimate of the success of the intervention if it were to be rolled out as part of standard smoking cessation service practice.

**Trial registration:**

Current Controlled Trials ISRCTN33031001. Registered 27 April 2012.

## Background

People who try to stop smoking typically experience urges to smoke, often described as cravings, frequently in response to environmental cues associated with smoking (such as drinking alcohol). These urges decrease in intensity and frequency with time. The key to stopping smoking is to resist these urges. Effective medication for smoking cessation reduces the intensity of the urges [1,2], and this is the likely mechanism of action.

There are three licensed medications for smoking cessation: bupropion, varenicline, and nicotine replacement therapy (NRT). Varenicline is a nicotinic partial agonist and it is therefore surprising to find that it is more effective than a full agonist, nicotine [3]. An investigation considered the possible mechanisms of action and compared varenicline, bupropion, and a placebo [4]. It found that varenicline reduced urges to smoke to a lower level than did bupropion. Furthermore, varenicline led to lower satisfaction from a lapse (smoking episode) after quit day than did bupropion. However, many other mood-related symptoms of withdrawal were of similar intensity. This suggests that a key mechanism of action of the smoking cessation pharmacotherapies, which relates directly to efficacy, is controlling urges and reducing satisfaction from smoking. Unlike NRT, varenicline is used for one to two weeks prior to quit day, which might explain its superior efficacy. Using NRT while smoking reduces satisfaction from smoking [5] and so it seems logical to examine whether NRT used prior to quitting could make smoking cessation more successful. This is known as nicotine preloading.

The mechanism above is widely assumed to underlie the apparent effectiveness of preloading. If true, it should work independently of whether or not a person uses pharmacotherapy after cessation, or the type of pharmacotherapy used. The proposed mechanism is essentially that nicotine preloading reduces satisfaction from smoking, and this begins to undermine the learned association between smoking and reward.

### Evidence from systematic reviews and meta-analyses

Two previously published systematic reviews of nicotine preloading [6,7] reported very positive results, with Shiffman and Ferguson [7] giving an odds ratio (OR) of 1.96, 95% confidence interval (CI): 1.31 to 2.93 for six weeks abstinence, and an OR of 2.17, 95% CI: 1.46 to 3.22 for six months. The Cochrane review [6] gave a risk ratio (RR) of 1.79 (95% CI = 1.17, 2.72 for long-term abstinence (six or 12 months).

In preparing the application for the reported trial, we undertook an updated meta-analysis [8]. This meta-analysis also investigated three hypotheses to examine the possible mechanisms of action and explain the variation in results observed in the existing clinical trials. We included four more studies than previous reviews and the evidence was based on a meta-analysis of 2813 participants. The main findings of this review are as given below.

Our review showed much less evidence of efficacy than the earlier reviews. There was a weak, positive, but non-significant effect of preloading versus placebo and/or no treatment on short-term abstinence (RR = 1.05, 95% CI = 0.92, 1.19, *P* = 0.49), with marked heterogeneity (I^2^ of 69%, *P* = 0.002). The effect on long-term abstinence gave a slightly larger but not significant RR of 1.16 (95% CI = 0.97, 1.38), with less heterogeneity (I^2^ = 36%, *P* = 0.14). Indirect comparisons, however, suggested that longer-acting NRT (such as a nicotine patch) might be more effective than shorter-acting types (such as a nicotine gum and/or lozenge); RR for short-term cessation using a patch was 1.17 (95% CI = 1.00, 1.37), and for a gum and/or lozenge was 0.82 (95% CI = 0.66, 1.02, *P* = 0.009) for the difference in RRs. For longer-term cessation the RR were (for patch) 1.26 (95% CI = 1.03, 1.55) and, for short-acting NRTs, 0.87 (95% CI = 0.60, 1.26), although the difference between the sub-groups was not statistically significant (*P* = 0.09). There is good evidence that smoking on a patch leads to higher blood nicotine concentration than from smoking alone. However, smoking while using short-acting NRT leads to concentrations similar to that from smoking alone [9]. This difference in response to smoking, while using these types of NRT, might explain the apparent difference in efficacy between patch and other NRT.

Second, we examined whether there was evidence to suggest that preloading works because it reduces positive or negative reinforcement from smoking and there was modest support for this. One study [5] reported reduced reward and one trial [10] reported no effect on positive reinforcement. Four studies [5,10-12] reported data relevant to negative reinforcement of smoking (feeling the need to smoke to stave off withdrawal) and none found evidence of this effect. However, we would expect that reduced reinforcement of cigarettes or reduced need to smoke should result in reduced consumption and this was observed. In five studies [5,10,11,13,14] where participants were asked to smoke as they wanted, there was a variable reduction in cigarettes per day, with a smaller and variable reduction in biological markers of smoke intake. One study, Schuurmans *et al*. [12], asked participants not to change their smoking and little reduction in consumption was noted. Studies in which participants were asked to reduce consumption showed the largest decline in consumption [15,16]. These studies used short-acting NRT to support reduction, which showed no evidence of efficacy over standard NRT use. The final part of this potential pathway is that reduced reinforcement from smoking leads to reduced withdrawal after cessation, but six studies showed no evidence of this [5,10-13,16].

Our second meditational hypothesis was that nicotine preloading worked because it enhanced adherence to post-cessation NRT. In all trials [5,10-16], nicotine preloading was followed by nicotine post-cessation pharmacotherapy. There is good evidence that adherence to NRT (patch or short-acting) after quit day enhances cessation [17,18]. Four studies reported data and none of these showed enhanced adherence to nicotine replacement therapy in preloading participants post-cessation [12,14-16]. As two of these studies were ‘positive’ studies [12,14], this is good evidence that NRT preloading does not enhance cessation through enhanced post-quit day adherence to NRT.

Our third hypothesis was that preloading enhanced confidence in quitting, which has been shown to be moderately associated with enhanced cessation success [19]. Two studies [15,16] reported contradictory data, but this indicates no good evidence to support this hypothesis.

We concluded that there was insufficient evidence to recommend preloading as a strategy for use routinely and that further trials were needed to confirm effectiveness. The best supported hypothesis was that preloading may work by altering desire to smoke. The trials were heterogeneous in ways that defied easy explanation. This raises the possibility that there is true heterogeneity in response to preloading with nicotine patches. Some people may benefit and others may not benefit from preloading.

Investigating mediation is important. Variation in response to the mediator is likely to give a much clearer signal about the efficacy of a particular strategy for preloading than is longer-term abstinence, and it has important practical implications for treatment. Instituting preloading in the stop smoking service (SSS) would cost approximately £50 million. If preloading was effective for only half of users, it would be useful to know which half. If we could monitor response to treatment (monitor a mediator), then we might be able to stop preloading in patients who are not responding, saving tens of millions of pounds. Alternatively, we could use the patient’s response measured by the mediator to adjust treatment (such as dose, duration, or form of medication) to enhance efficacy.

### Rationale for current trial

A further trial of nicotine preloading is required to improve the precision of the estimate of effect of preloading, to try and establish the cause or causes of the heterogeneity in the current trials, and to enhance understanding of the mechanisms and moderators of action. Preloading will be defined as four weeks of pre-quit patch use; as our review [8] found that patch was more effective than short-acting NRT, and based on the potential mechanisms of action proposed above, longer preloading (four weeks) is likely to be more effective than shorter preloading (two weeks). So far only one small study (N = 80) has tested the effect of four weeks of nicotine patch preloading [10].

### Rationale for a control intervention

The control arm of this trial will not receive a placebo treatment as the funder was keen for this trial to be pragmatic and reflect the effect of the intervention as it would be carried out in practice. This could lead to bias, as participants in the control arm may feel that they are not receiving an intervention and therefore be less likely to continue in the trial after randomization. This could lead to differential dropout between groups. Additionally, a lack of treatment in the control arm could mean that participants receive less contact or interaction with the researcher, which itself may boost success. In order to counteract this potential bias and to engage participants in the control arm, we propose a minimal intervention in the control arm, comparable to the intensity of the preloading treatment, but unlikely to influence effect.

### Trial objectives

To examine the relative effectiveness of nicotine patches worn for four weeks prior to quitting plus standard NHS care post-quit versus standard care only in smokers undergoing NHS treatment for tobacco dependence; To examine the safety of the nicotine pretreatment; To examine the incremental cost-effectiveness of nicotine pretreatment; To examine possible mediating pathways between nicotine pretreatment and outcomes; To examine moderators of the effects of preloading, including demographic characteristics, previous use of pharmacotherapy to quit, smoking history, and baseline levels of dependence;. To investigate opinions of the preloading intervention; To assess adherence to preloading treatment and subsequent standard smoking cessation pharmacotherapy.

## Methods/Design

### Plan of investigation

This is an open-label pragmatic randomized controlled trial to compare 893 motivated to quit smokers using a determined dose of nicotine patches for four weeks prior to quitting, with 893 of the same type of participants randomized to a control group of standard NHS treatment, with no placebo.

### Trial outcome measures

The primary outcome is six-month prolonged abstinence, measured by the Russell standard criteria [20], defined as a grace period of two weeks, followed by smoking fewer than five cigarettes thereafter and biochemically confirmed by an exhaled CO of <10 parts per million (ppm) (primary outcome). Other measures of abstinence are Russell standard four-week and 12-month prolonged abstinence and seven-day point prevalence biochemically confirmed abstinence at four weeks, six, and 12 months (secondary outcomes). We will assess adverse events related to NRT patch use and symptoms of nicotine overdose (such as nausea or excessive salivation) at each contact. We will estimate the costs of behavioral support and NRT, in order to calculate cost/lifetime quitter, the cost/life year gained and the cost/quality adjusted life year, and health service use. We will measure potential mediators of the preloading effect, such as changes in expired air CO between baseline and quit date, aversion and/or nausea, dependence, ratings of smoking reward, urges to smoke, stereotypy, confidence in quitting, and motivation to quit. We will examine potential moderators of the preloading effect, including demographic characteristics, previous use of pharmacotherapy to quit, smoking history, and baseline levels of dependence. Participants will rate the helpfulness, whether they would recommend preloading, and other views about the intervention. We will record adherence to preloading in pre-quit period, and adherence to additional standard smoking cessation medication.

### Participant entry

#### ***Sample size***

**Figure 1:**
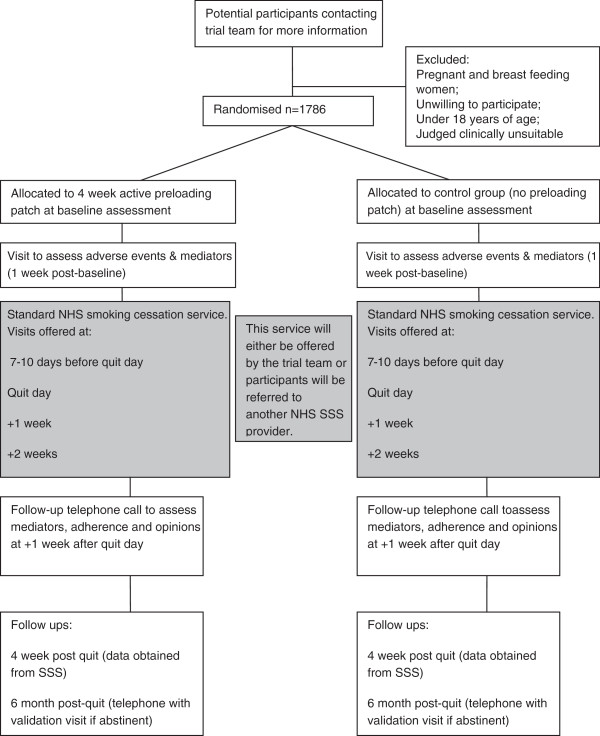
Diagram of planned participant flow through the trial.

893 participants in each of the two treatment groups; 1786 in total. See Figure 1 for an illustration of participants’ planned flow through the trial.

### Recruitment

We propose recruiting people in the following ways:

Recruitment centers at the Universities of Birmingham, Bristol, and Nottingham (United Kingdom) will recruit through general practitioner (GP) surgeries. Surgeries will be asked to write to patients who are listed as smokers on their clinical database, asking them to call the research team if they are interested in participating in the trial. The letter will encourage the person to stop smoking and to take the opportunity to enroll in the trial as a means of doing so. The content of the letter is therefore similar to a conversation between a GP and a patient. In some practices, as well as or instead of sending letters (depending on the circumstances within the practice), we will ask practices to send out text messages to smokers advertising the trial using their text messaging system, and will ask them to give out fliers advertising the study with patient’s repeat prescriptions. Again, interested patients will be provided with the contact details of the trial team. Additionally, we will give GPs referral cards detailing the telephone recruitment number to give to their patients who smoke, display advertising posters in GP practices, and provide GPs with mouse mats reminding them about the trial and to refer participants who are smokers. Screening will take place when a potential participant contacts the trial team to express interest in taking part.

To supplement recruitment where necessary these three centers will also ask NHS health services such as GP practices and NHS SSS, and community venues, if they have rooms for the research team to use to conduct clinics to recruit participants from a wider area. This will offer centralized clinic locations, and so will allow for a wider range of advertising possibilities. We will advertise for smokers to join the trial clinics held in these central clinic locations using newspaper, magazine, and/or internet advertisements, posters and fliers advertising the study distributed and displayed within the community. The study will be advertised in or alongside payslips distributed at the recruitment centers. SSS will be asked to write to people who have used their services in the past and are believed to still be smokers, inviting them to take part in the trial. Researchers will seek agreement from individual SSS, to inform smokers booked to attend the service about the possibility of taking part in the trial before their first appointment. Researchers will then enroll anyone who wishes to take part and refer them back into the SSS after their trial treatment (preloading or coping treatment). Recruitment centers are running a number of studies in their university departments. In cases where participants would be appropriate to take part in the Preloading Trial (adult smokers not already quitting), participants will be provided with a referral card or leaflet advertising the trial.

At the additional recruitment centre (Queen Mary University of London, United Kingdom) participants will be recruited at an existing smoking cessation clinic which accepts self-, primary care-, and secondary care- referrals. This final centre will offer patients who present to this clinic for treatment the chance to participate in the clinical trial, and will act as a site. Stop smoking services commonly advertise for patients. Therefore, at this site we will also use advertising as necessary to supplement recruitment into the trial, as would usually take place.

### Pre-randomization evaluations

Potential participants will telephone the trial office for further details. Office personnel will give information about the study, and if the potential participant wants to be screened for eligibility, will then use the online database to assign them an identification number and perform preliminary eligibility screening. We will only ask potential participants to provide personal contact details if screening is successful. We will then send the potential participant a patient information sheet and details of an appointment at a clinic to attend for fuller discussion with the researcher, signing the consent form, further screening, and trial entry procedures. In three of the four centers, we will use the participant’s own GP surgery, local SSS clinic, or a community clinic location as the venue for the initial meeting and this may or may not be the venue where the participant receives NHS smoking cessation support, depending on the surgery or service. In the fourth centre, the initial meeting will take place in the London-based smoking cessation clinic.

To be recruited onto the trial participants need to fulfil the inclusion criteria stated below, assessed on telephone screening and at the baseline clinic visit:

### Inclusion criteria

We will include smokers (defined as regular smokers of cigarettes, cigars, and tobacco cigarettes combined with marijuana) aged ≥18 years of age, who, in the judgment (see below for how judgment shall be made) of the trial researcher, would be suitable for preloading. Smokers must be seeking NHS support to stop smoking and willing to quit in four weeks. They need to be able to understand and consent to, and willing to comply with, study procedures.

### Exclusion criteria

We will exclude pregnant or breastfeeding women, people with extensive dermatitis, other skin disorder, or other severe adverse reaction that precludes patch use. We will exclude people who have had an acute coronary syndrome or stroke within the past three weeks. We will also exclude people with active pheochromocytoma and uncontrolled hyperthyroidism.

The judgment of suitability for preloading will aim to include more addicted smokers and exclude smokers with such low levels of addiction that the preloading patch may cause unacceptable toxicity. It will be based upon the time to first cigarette in the morning with earlier use reflecting higher addiction; number of cigarettes smoked per day with a greater number reflecting higher addiction; higher exhaled CO, which reflects higher addiction; and failure of previous quit attempts despite use of appropriate pharmacotherapy.

As all recruiting researchers will be trained stop smoking advisors, definitive cutoffs will not be used and researchers will use their judgment to decide suitability based on the combination of the criteria above. In cases of uncertainty researchers will be asked to use the following guidance and/or consult the appropriate principal investigator.

In addition to daily smoking, two of the following must be true: time to first cigarette after waking of less than two hours, exhaled CO more than five ppm, or failure of more than one previous quit attempt, despite use of appropriate pharmacotherapy.

### Withdrawal criteria

Should participants wish to withdraw from the trial, they will be given every opportunity. It is standard practice in smoking cessation trials to regard those who fail to attend for support and treatment as having relapsed, which is based on some evidence [20]. Therefore, failure to attend will not count as withdrawal from the trial and the only withdrawals will be those where a patient asks to be withdrawn. Such patients will not be replaced and, unless they refuse permission, data available up to that point will be used. Such withdrawals are expected in fewer than 5% of participants.

We will exclude from the trial all those who have had severe adverse reactions previously. Given the established safety profile of NRT and the evidence from studies of participants using NRT while smoking [9], we do not expect any serious adverse events due to the medication. Nevertheless, there will be a detailed work instruction for the trial that will detail the assessment of adverse events, and the procedure for defining and managing serious adverse events (SAE) and suspected unexpected serious adverse reactions (SUSARs). In the event of a SUSAR, or serious adverse reaction (SAR), the prescription for NRT will be withdrawn and not reinstituted in that person. The person will continue in the trial and be part of both the safety and effectiveness populations.

### Randomization and enrolment procedure

#### ***Randomization or registration practicalities***

Participants shall be randomized to a treatment arm at their baseline visit. They will be randomized to the intervention or control (1:1 ratio) on the basis of a computer-generated allocation sequence via the internet, with telephone backup, which will be provided by our electronic Primary Care Research Network (ePCRN). This will incorporate an online case report form (CRF) that, when basic details have been completed, will accomplish randomization. We will block randomize participants, stratified by centre, to account for the differences in recruitment and treatment delivery between the London research centre and the remaining centers. For very rare occasions when access to the network, and therefore database randomization is not available, we will have a backup process involving sequentially numbered, opaque, sealed envelopes for randomization. Each researcher will hold a small number of envelopes, to be used as a last resort only.

It is common for two people, often partners, to want to quit together. There is no imperative for the couple to be given the same treatment as one another, as the absence of a placebo means mixing up medication is unlikely, and not randomly allocating a person to the same intervention as their partner may introduce clustering effects. Therefore, in these cases we will consent for each member of the couple to be randomized individually, and will randomize each individually using the standard allocation procedure used for all other participants.

### Study procedures

#### ***Trial treatment providers***

The provision of participant treatment in the trial shall be carried out by researchers from both a clinical and non-clinical background. All shall be trained by the NHS SSS and the National Centre for Smoking Cessation and Training (NCSCT) to provide stop smoking support and to advise and provide all pharmacotherapies available through the NHS SSS. This is the maximum equivalent to what is required for NHS SSS advisors. In addition to this all researchers will be provided with trial specific and Good Clinical Practice (GCP) training. However, in the case of any uncertainty (regarding suitability for inclusion) researchers will be able to contact the principal investigator (PI) for the relevant site, for clarification by telephone. If the PI for the relevant site is not contactable then they shall contact another of the trial PIs. PIs will only delegate tasks relevant to the researchers’ training and experience.

### Provision of standard NHS SSS support

In addition to the research visits carried out below, all participants will receive standard NHS SSS support and medication whilst enrolled in the trial. One of the centers involved in this study (Queen Mary University of London, London) hosts an existing NHS SSS clinic operating within the East London SSS. All participants recruited through this site will be provided with both trial support and treatment, and standard NHS SSS support and treatment by the research team. Therefore, participants will be booked in for standard support at the baseline trial visit. The remaining three centers will be recruiting through GP practices and local SSS across a number of trusts. In some trusts and practices it may be most favorable (where local funds allow and a service is not already provided within the GP practice) for the research team to carry out both the trial and standard NHS SSS procedures (acting on behalf of the local NHS SSS, as above), whereas for others it may be most favorable to refer participants to their local SSS for their standard treatment (where local funds do not allow and a local service convenient to the patient is provided). In which case the research contacts described below would be carried out by the research team and the standard support by the SSS. In cases such as this researchers will book participants into a local SSS clinic at the baseline research visit, and a link will be made with the local SSS requesting the date of quit day and four-week quit data for the participants concerned. In a previous trial this has been achieved by requesting reports from the SSS, however more and more services are using a centralized database, which the trial team will request access to. Consent will be requested from participants to access their SSS records. Where appropriate, where the study team will only be conducting research activities and will not be providing any standard NHS support, GP practices, and SSS may be asked to act as participant identification centers (PICs) only. In this case GPs will be asked to inform their patients who are smoking about the trial and ask if they would like to take part (as described previously), and in some cases asked to hire a room out to the study team, which will be used to carry out the research activity.

### Research contacts

#### ***Visit 1 - baseline - enrolment***

The purpose of this visit is to explain the trial and seek written consent for any further trial procedures. These includes further screening for participant eligibility and randomization if the participant is eligible. The researcher will also collect basic data, take relevant samples, and dispense the preloading and provide support to enhance adherence to NRT (in the preloading arm only). Data to be collected by the researcher at this visit is reported in Table 1.

**Table 1:** Data collection to take place at the baseline visit

Demographics	Basic demographic information (date of birth, gender, ethnic group, educational qualification, occupational classification),
	Additional baseline measures to allow future assessment of predictive ability of variables on weight change from baseline to follow-up:, heaviest weight to date, weight gain. For more information on investigation of weight gain see Additional file 2.
Medical history	Medical problems and concomitant medication.
Healthcare usage data	Baseline healthcare use (including primary care and secondary care visits) for economic analysis.
Mediators	Baseline measures to allow future assessment of change in potential mediators of the preloading effect, such as dependence, nausea from smoking, reward from smoking, urges to smoke, smoking stereotypy, confidence in quitting, motivation to quit.
Past smoking and quitting history	Information on smoking history (cigarettes per day, age at commencement, dependence, longest period of previous abstinence), and exhaled CO.
	Additional baseline measures to allow future assessment of predictive ability of variables on weight change from baseline to follow-up: cigarettes per day, alcohol intake, previous quit attempt.
	Previous use of pharmacotherapy for cessation and experiences of doing so, to assess suitability for treatment, and to examine whether it moderates the effectiveness of preloading.
Biological samples and measurements	Blood sample to identify genetic information (this will only take place at research centers with the resources to do so, where researchers are trained in phlebotomy, and will be optional for participants). For more information on genetic investigation see Additional file 1.
	Weight, height Participant weight recorded using self-report and weighing scales. As people who relapse will not attend clinic at 6 and 12 month follow-ups the difference between self-reported and measured weights will be applied as a corrective adjustment to self-reported weight at follow-up in these participants. For more information on investigation of weight gain see Additional file 1.
	Salivary sample to measure cotinine concentration (the best measure of smoking intensity).

#### ***Visit 2 - one week after enrolment***

The purpose of this visit is to examine adverse events to see whether they are due to preloading in accordance with GCP, to collect a measure of exhaled carbon monoxide (CO), to assess adherence to preloading in the preloading trial arm, to collect a salivary sample for cotinine measurement and measure other potential mediators (as on visit 1). If this visit reveals problems with adverse events or requires alteration of the dose of the preloading patch, further visits and/or telephone calls will be scheduled as seems appropriate to the researcher or PI.

In the control group, this visit has no therapeutic or safety purpose and is there solely for us to collect data. We therefore propose to compensate all participants for returning for this visit with £15 for travel expenses and the time involved.

Every effort will be made for this visit to take place exactly one week after baseline; however in cases where this is not possible, visit 2 will take place between week -3 and week 0, before data is classed as lost to follow-up.

#### ***Telephone call one week after quit day***

During this telephone call from the researcher to the participant, potential mediators will be measured (as at visit 1), as well as adherence to the nicotine patches during the preloading period, and opinions of the preloading intervention.

Every effort will be made for this contact to take place exactly one week after each participant’s quit day; however in cases where this is not possible this contact will take place between 5 days after quit day and week 4, before data is classed as lost to follow-up.

In some cases a participant may reach their quit date and fail at this attempt. In this case participants may reset their quit date. However as per NHS SSS guidelines this reset quit date would be classed as a new quit attempt by the service. Therefore, for the purposes of this study the original quit date will be classed as the quit date, and this date will inform follow-ups.

#### ***Four week follow-up***

As described above, in some cases quit data from this follow-up will be collected by the research team, however in other cases it will be collected by SSS operating outside of the trial. In the latter case we will set up a system with the local SSS to gain access to this data. Smoking abstinence at four weeks is defined as no smoking at all in the past two weeks, confirmed by CO <10 ppm.

#### ***Six month telephone and clinic follow-up***

We will telephone participants six months after their quit day to establish abstinence and measure relevant mediators. Participants who declare abstinence will be invited to return to clinic for exhaled CO measurement to confirm this. As this is not a therapeutic visit, participants returning for the visit will be compensated £15 in lieu of travel expenses.

Every effort will be made for this contact to take place exactly six months after each participant’s quit day; however in cases where this is not possible this contact will take place between two weeks prior to six months post-quit and month nine, before data is classed as lost to follow-up.

#### ***12 month telephone follow-up***

All measures as for six month follow-up and participants attending will be compensated £15 in lieu of travel expenses.

Every effort will be made for this contact to take place exactly 12 months after each participant’s quit day; however in cases where this is not possible this contact will take place between two weeks prior to 12 months post-quit and month 15, before data is classed as lost to follow-up.

At the time of booking baseline, 6 month and 12 month clinic visits participants will be sent an appointment letter with the date, time and location of their appointment. In addition participants will be sent a reminder text 24 hours before each clinic visit (visit 1, visit 2, 6 month, 12 month) to help to ensure that they remember the appointment and maximize follow-up rates.

### Treatments

#### ***Treatment arms***

##### 

**Intervention** The active intervention is a 21 mg Niquitin CQ Glaxo Smith Kline Consumer Healthcare (Beecham Group PLC, 980 Great West Road, Brentford, Middlesex, TW8 9GS, United Kingdom) nicotine patch. Participants will wear these for four weeks before their smoking quit day, from the day of their enrolment. They will be advised to wear the patch for 24 hours a day. Participants will be advised to smoke as normal and avoid reducing consumption, during pre-quit patch treatment. Allowing nicotine concentrations to fall may mean cigarettes will be more rewarding, undermining suspected mechanisms [21]. We will help participants plan to keep to their consumption, for example by asking a 20-per-day smoker to make sure they empties their pack by bedtime if possible. However, participants will be free to reduce and not pressured to smoke if they find this difficult.

Although we aim that participants shall preload for four weeks, in some cases we will need to book participants into convenient NHS stop smoking clinics in order for them to also receive standard NHS smoking cessation support, and some participants defer their quit date. Consequently, participants will be booked into cessation clinics to seek to ensure a target quit date between three and five weeks after enrolment. In the event that a participant has not yet reached their quit date but wishes to delay it (in particular, this may occur in the case of personal difficulties that the smoker feels will seriously impair their chances of being successful) then the participant will be able to delay their quit date to a maximum of eight weeks following their baseline appointment, and will receive a maximum of eight weeks’ worth of nicotine patches for preloading.

The manufacturer, GlaxoSmithKline plc (GSK) (T/A GlaxoSmithKline Consumer Healthcare, Brentford, TW8 9GS) will deliver the medication to the trial centers. This is an open-label RCT where medication is dispensed in clinics operating within the NHS and therefore there are no special labelling or packaging requirements. Medication will be labelled and packaged as for normal clinical use and stored at the centers in facilities that meet the requirements of GCP. A risk assessment will take place to check the facilities for storage of the medication, to ensure that the drug will remain stable and will be stored securely, on a centre by centre basis. If the risk assessment deems it necessary we shall keep temperature logs to monitor the environment in which the medication is kept. The researchers (trained in smoking cessation treatment and medications) will dispense the medication in accord with the protocol and will record the batch numbers on the CRF, as is common practice in the NHS. Each person will usually be dispensed two weeks of patches initially, which is sufficient to cover treatment to the second visit but allows extra should that visit be missed. At the second visit (one week after baseline), we will usually dispense three more weeks of medication to allow up to five weeks of preloading. The researcher will be able to use some discretion when dispensing the number of patches needed, for example to allow for cases where participants undertake a lot of exercise and so patches are likely to fall off and need replacing more commonly. The participant will not pay a prescription charge for the medication as the medication will be donated free of charge by GSK.

If participants have patches left over when they reach their quit day and they are not using nicotine patches as part of the standard NHS post-quit treatment we will encourage them to use up their patches after quitting. The rationale behind this is that otherwise we will be asking participants to stop using their patches when they are most vulnerable (on their quit day), which could contribute to failure to quit.

##### 

**Control** This trial will be open label, and the comparator to preloading will be standard stop-smoking treatment, with no other intervention. After the participant is randomized to no intervention, they will begin a four-week period prior to their quit date where NRT will not be used to allow comparison with the intervention arm. Participants will not be advised to change their smoking behaviour in any way. The control arm will also be referred to standard smoking cessation support at the first visit, where necessary.

##### 

**Trial behavioral support** In both trial arms researchers will provide behavioral support. In the preloading arm, when preloading patches are dispensed at the baseline visit, the researcher will provide support - explaining the rationale as to why nicotine preloading might be helpful, how to use the patches, including helping participants to set reminders to use the patch, providing evidence on safety and tolerability of preloading, common side-effects and how to deal with them - as well as supply a booklet describing the rationale of preloading treatment, with the aim to enhance adherence. At the second visit, the researcher will enquire about participants’ understanding of the necessity of using preloading and ask about their concerns and address these as appropriate. In the control arm, participants will not be provided with this in-depth information about preloading, but will be provided with comparative counselling. This will involve asking participants to think about the cigarettes they smoke, what triggers these, and which they find most rewarding, and will be accompanied by a comparable booklet explaining the theory behind cue-associated learning.

##### 

**Standard SSS support** Standard smoking cessation support will not be altered in the intervention group by the previous preloading. Support typically commences one to two weeks prior to a target quit date and provides behavioral support on quit day, and then weekly until four weeks afterward. This support addresses issues such as planning for the quit day, the ‘not a puff rule’, and how to deal with difficult situations. It also provides monitoring of behaviour and validation of abstinence through CO testing. SSS providers have training to provide this behavioral support, largely modelled on withdrawal-orientated therapy [22]. This behavioral support for cessation will begin two to three weeks after the commencement of preloading treatment so that the target quit date is three to four weeks after commencement. Pharmacotherapies provided as part of this support are either NRT, varenicline, or bupropion and SSS involved in the trial will be informed that they should provide these to participants as is usual.

### Dose modifications for toxicity

Twenty-four hour patches can lead to problems of night time wakefulness or vivid dreams. Participants will be warned of this and anyone who has suffered from this in the past (assessed at baseline) will use the patch for 16 hours per day initially. In addition, we will warn participants to use the patch during daytime only should they suffer from difficulty sleeping. There is no evidence that 24 hour patches are more effective than 16 hour patches for cessation [6] and no reason to assume that the effectiveness of preloading depends upon 24 hour wear. NRT has been shown to be safe and there are no plans to modify the dose of NRT dispensed in the intervention group. However the dose of the patch may be reduced (to 14 mgif the participant reports previous experience of adverse reactions to a 21 mg patch and is not prepared to start 21 mg patches, the participant has symptoms of nicotine overdose (these include nausea, increased salivation, and pounding heart), or the participant wishes to reduce the dose because of presumed adverse effects attributed to the patch.

The intervention will be stopped and not reinstituted if the participant no longer wishes to use the preloading and/or decides not to proceed to a quit attempt, clear symptoms or signs of nicotine overdose are observed, not remedied by reduced dose of patch or reduced smoking (these include nausea, increased salivation, and pounding heart), the participant has some intervening health state that makes continued intervention impossible, for example admission to intensive care unit, a contraindication to this kind of NRT use or exclusion criterion emerges, for example the participant discovers she is pregnant.

The intervention may be temporarily halted and restarted. This may occur if a participant has an intervening health or emotional crisis, such as admission to hospital as an emergency, or a bereavement. It is likely that an intervening period of smoking off the patch is likely to remove any benefit from prior preloading. In this circumstance, the participant could choose to start the preloading again, which will be allowed once. For the purposes of counting abstinence, the quit day will be deemed to have been reached a maximum of eight weeks after the baseline visit, even if the participant is continuing with preloading and has not reached quit day at this point. If we lose contact with a participant and they do not make a quit attempt, this will not count as temporary halting of preloading and for the purpose of counting abstinence their quit day will be deemed to have taken place four weeks after the baseline visit.

In cases where the participant quits before their planned quit day, this day will be classed as the day they actually quit and the timing of follow-ups will be informed by this date, rather than the date originally planned.

### Concomitant medication

All medications will be permitted for use concurrently with preloading except those that are proven to help smoking cessation (bupropion, nortriptyline, mecamylamine, reserpine, and varenicline). These will be permitted for use in the latter part of preloading in preparation for a quit attempt, but not throughout preloading. The NHS clinic will either prescribe or arrange prescription of one of three first line smoking cessation pharmacotherapies: bupropion, varenicline, or NRT (used as a single form or combination NRT) at the dose that they see fit. Bupropion and varenicline should normally commence no sooner than two weeks and at least a week prior to the quit day initially set by the SSS as is standard, and standard NRT use commences on quit day. The choice of medication will be determined by the smoking cessation advisor and patient in consultation and bearing in mind guidance from the English body that sets guidelines (NICE) on choice. The NHS medication can continue for as long as the cessation advisor prescribes, with no special restriction imposed by the trial protocol.

NICE guidance advises against concurrent use of NRT and varenicline. However, this is due to the illogicality of the combination in normal post-cessation support, rather than evidence of safety concerns [23]. Concurrent use will have to happen in this trial if preloading is followed by varenicline post-cessation support. It is possible that NHS personnel would be more inclined to prescribe varenicline in the control arm than in the intervention arm, especially as patients may be comfortable and ‘responding’ to NRT. We will counter this in several ways. We will give a letter to the participant to give to the SSS therapist to explain the trial and to encourage free use of medication including varenicline. Second, we will monitor this issue and investigate corrective actions if we see it happening with particular SSS therapists or services. Third, we have addressed this in the analysis plan by proposing a sensitivity analysis to adjust for post-cessation medication use.

Data on all concomitant medication will be recorded. There is no special dietary or lifestyle advice that is imposed by using NRT and the associated regimens for using it proposed in this protocol.

### Trial management

#### ***Trial steering committee***

We propose a trial steering committee (TSC) with academic, primary care practitioner, and independent members. We will also incorporate a volunteer from a smokers’ panel group. The UK Centre for Tobacco and Alcohol Studies (UKCTAS) has a smokers’ panel to give smokers’ views on research priorities and projects. We will also incorporate an NHS service manager, to give NHS service views.

### Data monitoring committee

Following guidance on open-label and low-risk trials, we have agreed with the funder (National Institute for Health Research, Health Technology Assessment program (NIHR HTA)) that a data monitoring committee is not necessary.

### Pharmacovigilance

For information regarding the assessment of participant safety and reporting procedures please see Additional file 1.

### Assessment and follow-up

#### ***Assessment***

The primary and secondary outcomes will be measured as detailed in the previous ‘Trial design’ section. Further outcomes shall be assessed as follows:

At baseline, the researchers will collect the information stated in Table 1. Educational qualification and occupational classification will be classified in categories used in the UK Census 2011 [24], ethnic group will be classified according to categories used by the NHS SSS, based on the UK Census 2001, and exhaled CO will be measured using a CO monitor, as a measure of smoke intake.

Potential mediators of the preloading effect will be assessed at baseline, one week later during the pre-quit period, one week after quit day, and six and 12 months after quit day.

Dependence will be measured at baseline using the Fagerstrom Test for Nicotine Dependence (FTND) [25]. The FTND will be re-administered at the week following baseline visit, and at six and 12 month follow-ups. However, for the purposes of the analysis we will exclude the cigarettes per day item, which might reasonably be expected to decline in the active patch group without necessarily indicating reduced dependence.

Changes in reward from smoking, measured using the modified Cigarette Evaluation Questionnaire (mCEQ) [26], will be measured at all contacts (at post-quit contacts this will only be in those who have returned to smoking). We will ask the participant to focus on the cigarette after the evening meal (or equivalent in shift workers). For those who smoke after quit day, we hypothesize that preloading may result in decreased reward from smoking. The mCEQ measures satisfaction, taste, mood, cognitive, and sensory sensations to smoking particular cigarettes. At pre-quit contacts we will also use two simple, single item rating scales which provided more useful data than the mCEQ in our trial of varenicline preloading [27]. These ask participants to rate ‘Have you found your urges to smoke stronger or weaker than usual in the last week’ with response options of ‘Much stronger; slightly stronger; same as before; slightly weaker; much weaker’; and ‘Have you found cigarettes more or less enjoyable than usual in the last week?’ with response options of ‘Much more enjoyable; slightly more enjoyable; same as before; slightly less enjoyable; much less enjoyable’. We hypothesize that preloading would reduce satisfaction and the degree to which it does might be associated with improved outcome.

We will also measure changes in urges to smoke, measured using the urge strength and frequency questions from the Mood and Physical Symptoms Scale (MPSS) [28], at all contacts. These questions are both strongly correlated with the FTND, and predict successful smoking cessation more strongly than the FTND or other alternatives, such as the Questionnaire of Smoking Urges (QSU) [29,30]. Changes in stereotypy (a measure of the degree to which smoking is prompted by cues to smoke) will be measured using a subsection of the Nicotine Dependence Syndrome Scale (NDSS) [31], at baseline and the visit one week after baseline. Only two questions from this scale will be used as the other questions in the scale are either forced to change if cigarette consumption drops or could not be assessed over a short period.

Two variables that we do not expect to mediate the relation between preloading and abstinence will be measured. These are confidence in quitting and motivation to quit. They are often presumed to be mediators of smoking cessation success, but the effects have been found to be much less than supposed [19], so we will test this empirically. They are measured by single items only, using standard wording. These are: “How high would you rate your chances of giving up smoking for good at this attempt?” and “How important is it to you to stop smoking for good on this attempt?” (with response options of ‘Not at all; a little; somewhat; very; extremely, in both cases). These variables will be measured at baseline and after the first week of preloading.

Smoking consumption will be assessed at each contact using cigarettes per day, measured using self-report and/or changes in exhaled CO (to assess changes in smoke inhalation). This will be used to assess whether consumption reduces prior to quitting and to establish whether relapsed participants smoke less than at baseline or return to their previous consumption.

Aversion to smoking will be measured at baseline, the week following baseline, one week post-quit, and at six and 12 month follow-ups. Interviews with participants taking part in a previous trial [32] where participants were asked to use nicotine patches for two weeks whilst still smoking found that a number of participants thought that preloading worked because it made smoking aversive; for example by creating nausea and/or making a participant lose the will to smoke. This made some participants keen to quit. Therefore, we will test whether participants do find smoking aversive whilst using patches, and if so, for how long this effect persists. Interview participants’ responses suggest that markers of aversion are loss of the will to smoke and nausea. We are already measuring urges to smoke using the Mood and Physical Symptoms Scale [28]. We will measure nausea by asking participants to indicate the extent to which they have experienced nausea in the following circumstances: when they have seen cigarettes, lighters, or other smoking paraphernalia, and when they have smelt cigarette smoke. These situations were chosen as they are likely to be applicable to both participants who are smoking and those who are abstinent. Alongside this, at their visit one week after baseline, participants will be asked about the ease with which they are smoking alongside the patch. After being asked how many cigarettes per day they are smoking participants will be asked: ‘Have you had to force yourself to smoke these?’ (response choices: Yes or No), and ‘If so, to what extent?’ (responses range from ‘Very much so’ to ‘Not at all’).

We will assess the extent to which participants adhere to the preloading treatment at the visit one week after baseline and one week after quit day. As the time between these two visits will be at least a month participants will be given a simple means with which to record whether they have put their patch on each day. This will not be returned to the trial team but can be used as a memory aid by participants when reporting their adherence to the researcher. Potential side-effects of NRT patch use will also be measured at the contact one week following baseline assessment, and one week post-quit.

At the end of the first week after quit day, participants will be asked about their experiences of preloading. Their response will be recorded by ticking simple emergent categories that apply. These will be based on responses given in a recent interview study conducted as part of the Rapid Reduction Trial [32] and will include categories such as: ‘Did not feel urge to smoke’, ‘Smoking rate reduced’, and ‘Felt no effect of preloading’. They will also be asked to rate the helpfulness of the intervention, and whether they would recommend it to somebody else by answering the following questions: ‘How helpful did you find the preloading intervention?’ (answers rated on a scale from ‘Very helpful’ to ‘Not very helpful’), and ‘Would you recommend the preloading intervention to somebody else?’ (response choices: ‘Yes’ or ‘No’).

We will assess participants’ use of health services (including primary and secondary care) during the preloading and follow-up periods, so as to assess whether the intervention might have caused an increase in use (see cost-effectiveness analysis).

Researchers will obtain saliva samples to measure salivary cotinine concentration at baseline (while smoking only), and one week after enrolment (while smoking and using or not using nicotine patch). Following advice from the funder, funding for the analysis of cotinine samples is not available. We will seek this separately, when the study has been completed. If there is no effect of preloading then analysis of saliva samples will not be beneficial, and so will not proceed.

Observational investigations are also planned and are detailed in Additional file 2.

### Loss to follow-up

In accordance with the Russell Standard [20], we will conduct an intention-to-treat analysis, and assume those lost to follow-up are smokers. We will make three attempts to contact participants using their preferred method before an attempt at follow-up is abandoned.

### Trial closure

The end of the trial is defined as the last date of follow-up of the last patient, following database lock. However at present there is uncertainty as to when the last follow-up will take place. We will conduct a futility analysis with the aim of saving the resources of the funding body (NIHR HTA). The futility analysis will take place 30-months post the commencement of recruitment, or as soon thereafter as possible, when all the primary outcome data are collected. The analysis will examine the difference between arms in the frequency of occurrence of the primary outcome (six month follow-up data). If there is a lack of significant effect (*P* <0.05) at six month follow-up, then the 12 month follow-up shall be terminated and the study will close 36 months after commencement. If there is a significant difference in six month abstinence then 12 month follow-up will be completed, taking the study to 42 months duration.

### Statistics and data analysis

#### ***Sample size***

This is determined based on plausible estimates of the six month confirmed prolonged abstinence rate in the control group and the effectiveness of preloading. A recent trial showed a six month abstinence rate of 15% [33]. Another trial of 631 participants also found a similar prolonged abstinence rate [34]. We have therefore settled on an abstinence rate in the control group of 15%. Our meta-analysis found summary RRs of 1.05 for short-term and 1.16 for prolonged abstinence [8]. However, there was heterogeneity, perhaps explained by use or non-use of the patch, but other differences between trials make this uncertain. It is therefore difficult to settle on a specific RR, but we chose 1.4 as plausible and an effect likely to interest the NHS SSS. The RR (95% CI) for abstinence in the patch trials in our review [8], was 1.17 (1.00, 1.37) for short-term and 1.26 (1.03, 1.55) for long-term abstinence, but with unexplained heterogeneity, so our RR appears reasonable. For example, an RR of 1.4 means that the summary effect for NRT is about 2.2, similar to that for varenicline versus placebo (2.3) [3]. This gives us a sample size of 893 participants per arm or 1786 in total for 90% power, calculated with Yates correction using nQuery (Statistical Solutions Ltd, 4500 Airport Business Park, Cork, Ireland) (Table 2).

**Table 2:** Sample sizes required for different combinations of power and relative control versus intervention six-month abstinence rates

		**Trial with 80% power**	**Trial with 90% power**
**% prolonged abstinence in control**	**% prolonged abstinence in intervention**	**Number/arm**	**Number/arm**
RR = 1.3			
14	18.2	1249	1655
15	19.5	1150	1524
16	20.8	1064	1409
20	26	805	1065
RR = 1.4			
14	19.6	734	970
15	21	676	893
16	22.4	625	825
20	28	471	622
RR = 1.5			
14	21	490	646
15	22.5	451	594
16	24	416	549
20	30	313	412

### Analysis plan

The primary analysis will be performed using the full (intention to treat) dataset, including all those randomized, presuming that those who do not provide data at follow-up are continuing to smoke. We will compare the proportion of people achieving the primary outcome by calculating the RR and 95% CI. These figures allow clinicians to have an intuitive sense of the size of the effect. However, the primary effectiveness analysis will be based on an adjusted OR, calculating first an unadjusted OR (for comparison), and then an adjusted OR. We will adjust for two the precision of the treatment effect estimate, using multiple logistic regression in Stata (StataCorp LP, 4905 Lakeway Drive, College Station, Texas 77845-4512, USA) the length of previous abstinence achieved and baseline urges to smoke (using the urges questions from the MPSS), which have both been shown to be predictors of success [29,30,35]. In a sensitivity analysis, we will also adjust for post-cessation medication use, because varenicline is more effective and this might be imbalanced across treatment arms. Secondary outcomes will be analyzed similarly. *P* <0.05 will be considered statistically significant. We will calculate the proportion of people finding the intervention helpful, but not compare these between arms with inferential statistics.

The safety analysis will be performed on all participants who complete follow-up at one week following baseline or follow-up one week after quit day. We will examine the occurrence of moderate and severe AEs and SAEs, and compare between trial arms. We will also code events using the MedDRA coding database to examine the specific problems that might occur with preloading. We will relate these to baseline characteristics and changes in smoking behaviour, to investigate predictors of adverse events. The analysis will be performed in Stata using logistic regression.

The mediation analysis will proceed using the procedure outlined by MacKinnon, using regression modelling [36]. We will estimate the mediated effect using mediation regression equations, modelling the association between abstinence (dependent variable) and intervention, and between abstinence (dependent variable), the intervention and the potential mediators, in both cases using logistic regression. The association between (continuous) mediators and the intervention will also be modelled using linear regression. The mediated effect will be estimated by the product of coefficients method, using the appropriate coefficients from the regression models, and using boot strapping to compute confidence limits of the mediated effect. We also propose a two-step mediation process, whereby preloading leads to higher nicotine concentration (reflected by cotinine concentration), which in turn leads to reduction in measures of dependence that lead to improved abstinence. We will examine this using Mplus (Muthén & Muthén, 3463 Stoner Avenue, Los Angeles, CA 90066) with a structural equation model.

We will conduct exploratory subgroup analyses to examine whether the effect of preloading is similar for people who use varenicline or those using NRT (we expect the proportion using bupropion to be small), first testing for effect modification with a multiplicative interaction term in logistic regression. Similarly we will examine whether the effect of preloading is modified by dependence level (assessed by FTND, exhaled CO, and salivary cotinine), demographic characteristics, previous use of pharmacotherapy to quit, and smoking history. We will also investigate the proportions of reactors and non-reactors to the preloading treatment (those who do and/or do not experience reductions in CO, cigarettes per day and cigarette reward, between baseline and the visit one week following this), whether quit rates differ between these two groups, and whether this is modified by the pharmacotherapy participants are provided with from the NHS SSS.

We aim to address two aims concerning weight gain during a cessation attempt, details of the analysis plan for these can be found in Additional file 2.

### Cost-effectiveness analysis

Cost-effectiveness analysis will be conducted by combining data collected within the trial and existing models. We will calculate the proportion of prolonged abstinent participants produced by the intervention. We will estimate the costs of the behavioral support and NRT using a similar approach adopted in our previous HTA reports and economic modelling [37,38], using local costings where appropriate. These models will enable the calculation of the proportion of lifetime quitters, the cost/lifetime quitter, the cost/life year gained and the cost/quality adjusted life year. We will compare the health service use for the active and control groups to assess whether preloading leads to extra health service use. Longer term NHS costs can be estimated using a cost model recently developed by the Public Health Research Consortium (PHRC), involving one of the investigators [39]. Cost effectiveness acceptability curves will be used to demonstrate the value for money based on a range of threshold values for a quality-adjusted life year (QALY).

### Data handling, record keeping, and retention

The secure online database used for trial identification number allocation and randomization will incorporate an online CRF. There will however also be a paper copy of each CRF (the source documents for the trial). This is because the CRF will need to be completed to correspond with each clinic visit and follow-up; however there may not always be access to a computer or internet connection when trial clinics are taking place. In this case the paper CRF will be completed and data will be copied to the online version at a later date. CRFs will be kept in a locked cabinet in a secure office and department. These will be transferred from the site of the research visits to the universities personally by the researchers, and consent will explicitly be sought from participants to do so. The trial database will be securely developed, held and maintained by the Primary Care Clinical Research and Trials Unit (PC-CRTU) at the University of Birmingham. On completion of the trial and data checking, the CRFs will be transferred to a secure, GCP compliant, external archiving facility, where they will be held for 15 years and then destroyed. The database will be anonymized and a secure compact disc containing the link between identification number and patient identifiable information will be stored in a secure archiving facility.

The trial is being run as part of the portfolio of trials in the Primary Care Clinical Trials Unit (PC-CTU), a National Institute for Health Research (NIHR) recognized trials unit in Primary Care Health Sciences, at the University of Oxford. The data management will be run in accord with the standard operating procedures, which are fully compliant with the Data Protection Act and GCP.

Patient identifiable data will be shared only within the clinical team, on a need-to-know basis, to provide clinical care and ensure good and appropriate follow up. Patient identifiable data may also be shared with participants’ GPs and approved auditors from the REC, NHS Research and Development, or the MHRA. Otherwise, confidentiality will be maintained and no one outside the trial team will have access to either the CRFs or the database.

### Safety monitoring plan

#### ***Risk assessment***

A risk assessment has been carried out in accordance with MHRA guidance on Risk Adapted Approaches to Monitoring. A suitable monitoring plan will be drawn up and appropriate on-site and central monitoring will be performed.

### Monitoring at the study coordination centre

The trial will be conducted in accordance with the risk assessment, monitoring plan, current approved protocol, GCP, relevant regulations, and standard operating procedures.

Monitoring will be performed by the University of Oxford’s Clinical Trials and Research Governance (CTRG) Office according to CTRG standard operating procedures and GCP. Data will be evaluated for compliance with the protocol and accuracy in relation to source documents. Following written standard operating procedures, the monitors will verify that the clinical trial is conducted and data are generated, documented, and reported in compliance with the protocol, GCP, and the applicable regulatory requirements.

Data cleaning will take place by a series of logical checks on the electronic data (for example, a person cannot be recorded as a prolonged abstinent smoker at six months if they were not in such a state at four weeks). Discrepant records will be checked with the source documents and the database amended if necessary.

The trial may be subject to monitoring by the lead Comprehensive Local Research Network (CLRN). Direct access will be granted to authorized representatives from the Sponsor, host institution, and the regulatory authorities to permit trial-related monitoring, audits, and inspections. Therefore, participants will be asked for consent to allow their records to be viewed by these authorities.

### Monitoring at local sites

The monitor will perform site visits. The team will be trained in all aspects of the protocol and trial procedures, and the monitor will check this as a part of their visit (compliance to protocol, delegation logs and curriculum vitae).

### Serious breaches

The Medicines for Human Use (Clinical Trials) Regulations contain a requirement for the notification of ‘serious breaches’ to the MHRA within seven days of the Sponsor becoming aware of the breach.

A serious breach is defined as ‘a breach of GCP or the trial protocol which is likely to affect to a significant degree:

a) the safety or physical or mental integrity of the subjects of the trial; or

b) the scientific value of the trial’.

In the event that a serious breach is suspected the Sponsor must be contacted within one working day. In collaboration with the CI, the serious breach will be reviewed by the Sponsor and, if appropriate, the Sponsor will report it to the REC, MHRA, and the NHS host organization within seven calendar days.

### Regulatory issues

This study has CTA from the UK competent authority- the MHRA (CTA 21584/0322/001-0001) and has approval from the National Research Ethics Service Committee East Midlands - Leicester (REC reference: 12/EM/0014). The CI will ensure that this trial is conducted in accordance with the principles of the Declaration of Helsinki and with the ICH Guidelines for Good Clinical Practice (CPMP/ICH/135/95) July 1996. The CI shall submit once a year throughout the clinical trial, or on request, an annual progress report to the REC, host organization, and Sponsor. In addition, an end of trial notification and final report will be submitted to the MHRA, the REC, host organization, and Sponsor.

### Patient consent

The process for obtaining participant informed consent will be in accordance with GCP. The researcher and the participant shall both sign and date the consent form before any trial procedures begin.

A copy of the signed form will be kept by the participant, and the original will be retained in the appropriate site file. Another copy will be forwarded to the GP to file in the participant’s medical notes.

The participant’s decision to take part in the trial is entirely voluntary. It will be explained to potential participants that they can withdraw consent at any time without penalty or affecting the quality or quantity of their future medical care.

Participants will be informed of any relevant information that becomes available that affects their participation in the study. Revised consent forms will be used if applicable, and amended forms will be submitted to the main REC for favorable opinion prior to use. Revised informed consent forms will be signed by the parties specified above.

### Indemnity

The University of Oxford has a specialist insurance policy in place which would operate in the event of any participant suffering harm as a result of their involvement in the research (Newline Underwriting Management Ltd, at Lloyd’s of London, policy number: WD1200463). NHS indemnity operates in respect of any clinical treatment which is provided.

### Sponsor

The University of Oxford will act as the Sponsor for this trial. The Sponsor’s office can be contacted at: Clinical Trials and Research Governance, Joint Research Office, Churchill Hospital, Oxford, OX3 7LE.

### Funding

The NIHR HTA program is funding this trial. The application for funding was made in response to a themed call by the HTA program.

### Audits

The trial may be subject to inspection and audit by the University of Oxford (under their remit as Sponsor), the study coordination centre and other regulatory bodies, such as the MHRA, to ensure adherence to GCP.

### Financial arrangements

#### ***Participant payments***

Participants will receive payment for travel and inconvenience at the following visits: one week post-enrolment - £15 to intervention and control groups, Six month clinic follow up - £15 to intervention and control groups, and 12 month clinic follow up - £15 to intervention and control groups.

### GP payments

No payments will be made to GP surgeries or NHS SSS providers, aside from NHS service support costs, as agreed with the Primary Care Research Network (PCRN).

## Discussion

The proposed study is warranted due to the uncertainty of the effect of nicotine patch preloading. The RCT design is a strength, as it means that we can maximize the likelihood that the differences observed between groups are due to the intervention rather than potential confounders. Blinding participants and researchers, and using a placebo control would have increased this likelihood; however the use of a pragmatic design, which mimics how the preloading intervention would be carried out in practice, means that resulting relative estimates of effectiveness will more accurately represent what we would expect to see if the intervention were carried out in a real-world setting. The use of an intention-to-treat analysis also helps to ensure this; not all participants will adhere to the treatment recommended in their allocated study arm, however this is what we would also expect to see in practice. Excluding those participants who do not adhere to treatment from the analysis may produce a biased estimate of effectiveness. An intention-to-treat analysis also assumes that all those participants lost to follow-up have returned to smoking, which is standard practice in smoking cessation trials; however we will report and assess attrition rates and compare these between trial arms to investigate whether there appears to be any factors associated with attrition. We will also assess whether the assumption that those with missing data are smokers affects study findings by exploring alternative associations between missing data and the odds of smoking [40]. Another strength of this study is that it aims to recruit a large number of participants (N = 1786), and so will have the power to detect useful differences in effect between usual smoking cessation support and the addition of nicotine preloading treatment to this support, which no other previous trial has been able to do. It will provide a significant contribution to the existing pool of data in this area.

However, a potential barrier to recruiting such a large sample is unexpected low response rates to recruitment strategies. For this reason, although there is a core recruitment strategy (sending letters from GP practices to smokers and recruiting participants through a smoking cessation clinic), other potential recruitment strategies are outlined in the protocol that can be utilized where necessary, such as using community and online advertising mechanisms to recruit participants from the wider community. The use of several recruitment centers mean that centers can try different strategies and share expertise.

The length of follow-up (12 months) is also a strength of this study. Many smokers who make a quit attempt will relapse; therefore long follow-up periods are needed in order to gain a realistic impression of the impact that a smoking cessation intervention will have, in terms of long-term health and economic benefits. However, if a nicotine preloading intervention has not shown an effect at six months then there is no reason to believe that it will have an effect at 12 months, and so a futility analysis will be carried out using primary outcome data to establish whether a 12 month follow-up is necessary. This will prevent the use of unnecessary resources and reduce participant burden if there is no effect found on the primary outcome at six months.

Finally, we seek to maximize the return on investment by addressing several other questions in addition to the primary one, which is the effectiveness of preloading. However, our sample size calculation is based on the primary outcome, as is standard practice in trials such as this, and all analyses of additional outcomes will be interpreted as secondary. By addressing the mechanism of effect, we may be able to create tools that will allow therapists to assess whether preloading is effective for particular patients, as well as advancing the science of understanding nicotine addiction. In addition, by collecting data on participant weight and a blood sample for genotyping (see Additional file 2), we can investigate the impact of smoking cessation and relapse on weight change, predictors of this weight change and potential genetic predictors of smoking behaviors.

## Trial status

Recruitment began in August 2012. At the time this manuscript was submitted for publication the trial was still in the recruitment phase, with 1311 participants recruited as of the 12 June 2014. As response rates to letters from GP practices and the London smoker’s clinics advertisements have been lower than expected, these core recruitment strategies have been supplemented by the additional strategies listed in the ‘Recruitment’ section above. This manuscript is based on version 6.0 (30 July 2013) of the study protocol.

## Abbreviations

CI: Confidence intervals; CLRN: Comprehensive local research network; CO: Carbon monoxide; CRF: Case report form; CTA: Clinical trial authorization; CTRG: Clinical trials and research governance; ePCRN: Electronic primary care research network; FTND: Fagerstrom test for nicotine dependence; GCP: Good clinical practice; GP: General practitioner; GSK: Glaxo smith kline; HTA: Health technology assessment; ICH: International conference on harmonization; mCEQ: Modified cigarette evaluation questionnaire; MedDRA: Medical dictionary for regulatory activities; MHRA: Medicines and healthcare products regulatory agency; mg: Milligrams; MPSS: Mood and physical symptoms scale; NCSCT: National centre for smoking cessation and training; NDSS: Nicotine dependence syndrome scale; NHS: National health service; NICE: National institute for health and care excellence; NIHR: National institute for health research; NRT: Nicotine replacement therapy; OR: Odds ratio; PC-CTU: Primary care clinical trials unit; PC-CRTU: Primary care clinical research and trials unit; PCRN: Primary care research network; PHRC: Public health research consortium; PI: Principal investigator; PIC: Participant identification centre; ppm: Parts per million; QALY: Quality-adjusted life year; QSU: Questionnaire of smoking urges; RCT: Randomized controlled trial; REC: Research ethics committee; RR: Risk ratio; SAE: Serious adverse events; SAR: Serious adverse reaction; SSS: Stop smoking service; SUSAR: Suspected unexpected serious adverse reactions; TSC: Trial steering committee; UKCTAS: UK Centre for tobacco and alcohol studies.

## Competing interests

The pharmacotherapy used in the intervention arm of this trial (nicotine patches) is provided free of charge by GlaxoSmithKline plc. None of the authors or trial collaborators are directly employed by GlaxoSmithKline plc. NL-H has received consultancy fees and hospitality from manufacturers of smoking cessation/harm reduction products. PA, MM & HM have done research and consultancy for manufacturers of smoking cessation products. PH has received research funding from and provided consultancy for manufacturers of smoking cessation medications. DL has received hospitability from manufacturers of smoking cessation products, (Pfizer Ltd, Tadworth, UK). DL’s previous institution has received smoking cessation products for use in a clinical trial from Johnson & Johnson Ltd (New Brunswick, New Jersey, United States). DL has received Slimming World membership vouchers for use in an RCT for the prevention of cessation-related weight gain. DL has received expenses and consultancy fees from the NHS and Universities for teaching about cessation- related weight gain. DL has received grant funding from UKCTCS and the National Institute for Health Research - School for Primary Care Research (NIHR-SPCR) for research relating to cessation-related weight gain. AM has received travel funding, honorariums and consultancy payments from manufacturers of smoking cessation products (Pfizer Ltd, Novartis UK, and GSK Consumer Healthcare Ltd) and hospitality from North51 who provide online and database services. He also receives payment for providing training to smoking cessation specialists, receives royalties from books on smoking cessation, and has a share in a patent of a nicotine delivery device. In the last five years, TC has been paid to attend two research symposia arranged by Pierre Fabre Laboratories, a NRT manufacturer. GD, SL and SP have no competing interests.

## Authors’ contributions

NL-H, GD and PA drafted the manuscript and contributed to the design of this study. MM designed and drafted the genetics element of the study, contributed to the wider study design, and made comments on the manuscript. AM, HM, PH, SL and TC contributed to the design of this study and made comments on the manuscript. SP designed and drafted the health economics evaluation element of the study. DL aided in the design and drafted the weight change element of the study. All authors read and approved the final manuscript.

## Supplementary Material

Additional file 1 **Pharmacovigilance.** Further details on the assessment of adverse events. Click here for file

Additional file 2 **Observational analyses.** Details of secondary investigations into genetics and weight gain. Click here for file
