# Supplementary material for: Nicotine patch preloading for smoking cessation (the preloading trial): study protocol for a randomized controlled trial
Source: Trials. 2014 Jul 22;15:296. doi: 10.1186/1745-6215-15-296 (PMC4223826; doi:10.1186/1745-6215-15-296)
Supplement: Additional file 1 — Pharmacovigilance. Further details on the assessment of adverse events. [file 1745-6215-15-296-S1.docx]

# Additional file 1: Pharmacovigilance

## Assessment of safety

Potential participants’ safety will be ensured by screening for eligibility using a structured form completed by the researcher. This will record evidence of eligibility and exclusion criteria. The researcher will be trained to assess the presence of exclusion criteria and we will provide a more detailed description of the conditions listed to aid their identification. In addition, the researcher will make an assessment of current medical problems to assess for other complicating diseases. Any queries remaining as a result of this process will be resolved by discussion between the researcher, PIs and the relevant physicians providing routine medical care, usually the participant’s GP. Such concerns are unusual but not rare. Typically, they arise from a participant’s hazy knowledge or understanding of their past medical history, and are usually readily resolved. In addition, where recruitment takes place in GPs surgeries the researcher may have access to the participant’s medical record and therefore may be able resolve such issues promptly. No blood or further medical testing will be necessary to ensure safety.

NRT has been investigated in several hundred previous clinical trials, is widely prescribed worldwide, and is subject to safety monitoring. Thus, there is every reason to expect that treatment in this trial will be safe. Fagerstrom and Hughes’ review of smoking while on NRT concluded that “very few and mild adverse reactions were reported with concurrent smoking and use of NR[T], even when nicotine concentrations were elevated 2 or 3 times with use of very high doses of nicotine from patches”.p77[1] These were mostly short-term studies. A systematic review of long-term NRT and concurrent smoking found no evidence of an increased rate of death or serious adverse events.[2] As the cardiovascular response profile to high doses of nicotine (63mg patches) in smokers is flat, so there is no reason to assume that preloading poses problems for typical smokers.[3]

In the trials of preloading reviewed in our meta-analysis, there were no differences in the occurrence of possible side-effects or more serious problems between trial arms. Of these trials, Bullen reported that the overall occurrence of adverse events was 45% vs. 45% and of serious adverse events was 18% vs. 20% in preloading and non-preloading groups.[4] Rose 1998 reported dropouts from preloading treatment were due to practical difficulties.[5] Rose 2009 recorded the incidence of typical minor adverse health conditions (e.g. headache) and found the incidence similar in the preloading group and the placebo group.[6] Schuurmans reported the incidence of adverse reactions during preloading as 6% vs 2% (not statistically significantly different) in the placebo group- all mild skin reactions.[7] Therefore preloading is safe and well tolerated.

Participants will be warned about the side-effects of NRT and advised not to stop taking the medication without consulting with an NHS professional, preferably the trial team. To this end, all participants will be given the trial team’s contact details, which will allow participants to receive advice on medication or to report perceived serious adverse effects and receive advice on medication as required. Participants will record the occurrence of symptoms of nicotine overdose by completing a checklist at the one week post-baseline clinic visit. These will be given to the researcher, who will also enquire about any other adverse events, so as to determine the severity of any adverse event and ensure that appropriate advice is given for its management (such as rotating the patch site or use of emollients for skin reactions). Minor adverse reactions will be monitored and managed in this way. For each adverse event that we anticipate encountering the researcher will have a definition of clinical severity. For example, a mild skin site reaction to the patch will be defined as a burning sensation that does not interfere with normal activities, redness or swelling at the site of application, or mild blistering. Any reaction beyond that will be classified as potentially moderate or severe and will be reported to and discussed with the PI (or clinician delegated by the Chief Investigator (CI)). A decision on stopping therapy will then be made with the participant, attending clinician, PI (or clinician delegated by the CI), and other relevant parties as appropriate. In response to the nicotine overdose schedule the researcher will make an assessment of whether the NRT dose is too high or not, and then if relevant take action, such as continue with prescribed dose, direct the participant to use a lower dose, or reduce cigarette consumption. These actions will be recorded.

Nicotine has a short half life (2 hours), meaning that the blood concentration will not increase during the course of treatment, so that new side-effects are not expected after the first few weeks. In addition, reactions to it relate to local use such as skin discomfort from patches, and people become accustomed to these side-effects after a short time of using the preparation. At the clinic visit one week after baseline, participants will be advised to phone the contact number provided to report side-effects that occur from then on. The advice given will depend upon the severity of the reported reaction, and those with moderate reactions will be invited to an ad hoc consultation.

The Summary of Product Characteristics (SmPC) for the Niquitin CQ patch contain no warnings about SARs, except rare allergic reactions, such as angioedema, and cardiac arrhythmias, occurring in less than 1/1000 users. Thus, we expect no or very few SUSARs in this trial. The long history of nicotine patch use in and outside of trials for NRT means that SUSARs are unlikely. Participants will be given instructions for the reporting of SAEs. Therefore, through direct contact with the participant or contact from their attending physician, we expect to become aware of SAEs. Reporting procedures and definitions are as follows.

## Definitions

***Adverse event (AE)***

Any untoward medical occurrence in a patient or clinical trial subject, and which does not necessarily have a causal relationship with any treatment administered. An AE can therefore be any unfavourable and unintended sign (including an abnormal laboratory finding), symptom or disease temporarily associated with use of an investigational medicinal product (IMP) or in the same time period in the control group, whether or not considered related to the IMP*.* To be counted as an AE, an event must occur during the period from baseline to 1 week after quit day. Events that occur outside of the treatment period will be noted in a significant events log in the CRF, but will not be noted as an adverse event, as they will not be related to the treatment, and so would cloud the analysis if noted as such.

***Adverse reaction (AR)***

All untoward and unintended responses to an IMP, related to any dose administered. All AEs judged by the reporting investigator or sponsor as having a reasonable causal relationship to an IMP qualify as ARs. The expression ‘reasonable causal relationship’ means to convey in general that there is evidence or argument to suggest a causal relationship.

***Unexpected adverse reaction*** ***(UAR)***

An AR, the nature or severity of which is not consistent with the applicable product information (i.e. the SmPC). When the outcome of the AR is not consistent with the applicable product information, this AR should be considered unexpected. Side effects documented in the SmPC which occur in a more severe form than anticipated are also considered to be unexpected.

***Serious adverse event (SAE) or serious adverse reaction (SAR)***

Any untoward medical occurrence or AR that:

- Results in death;
- Is life-threatening – an event in which the subject was at risk of death at the time of event, not an event that hypothetically might have caused death if it were more severe;
- Requires hospitalisation, or prolongation of existing inpatients’ hospitalisation;
- Results in persistent or significant disability or incapacity;
- Is a congenital anomaly or birth defect.

Other ‘important medical events’ may also be considered serious if they jeopardise the participant or require an intervention to prevent one of the above consequences.

Medical judgement will be exercised in deciding whether an AE/AR is serious in other situations. Important AE/ARs that are not immediately life-threatening or do not result in death or hospitalisation, but may jeopardise the subject or require intervention to prevent one of the other outcomes listed above, will also be considered serious. For the purpose of this trial, SAEs will be those that occur after trial enrolment until one week after quit day. If the events described above occur, but not during this period, they will not be deemed SAEs. This is for the same reason as described in accordance with AEs. Planned hospital admissions that occur during this period will also not be recorded as SAEs, providing that the likely occurrence of these was recorded at the baseline assessment.

***Suspected unexpected serious adverse reaction (SUSAR)***

Any SAR related to an IMP that is both unexpected and serious.

## Causality

The assignment of causality will be made by the investigator responsible for the care of the participant using the definitions in Table 1. If in doubt about the causality, the local PI shall inform the study co-ordination centre, who will notify the CI. The pharmaceutical company and/or other clinicians may be asked to advise in some cases. In the case of discrepant views on causality between the PI and others, all parties will discuss the case. In the event that no agreement is made, the Medicines and Healthcare Products Regulatory Agency (MHRA) will be informed of both points of view.

## Reporting procedures

All AEs occurring from enrolment to one week post-quit shall be reported. Depending on the nature of the event, the reporting procedures below will be followed. Any questions concerning AE reporting should be directed to the CI in the first instance.

### *Non-serious AE/ARs*

All such events, whether expected or not, will be recorded in the paper CRF, and the electronic version of the CRF updated.

### *SAEs and SARs*

All SAEs occurring during the study, from enrolment to one week post-quit, either observed by the recruiting site PI or reported by the participant, whether or not attributed to study medication, will be recorded on the CRF and forwarded by the site to PC-CTU, using the “PC-CTU SAE Report Form”, following assessment for seriousness and relatedness by the PI. This form will be completed and faxed to the PC-CTU using the number quoted on the report form. The form should also be emailed to the PC-CTU using the email address quoted on the form. As a minimum, the following information will be recorded:

- Description;
- Date of onset;
- End date;
- Severity;
- Assessment of relatedness to study medication;
- Other suspect drug or device;
- Action taken;
- Follow-up information should be provided as necessary.

SAEs must be reported to the PC-CTU within 24 hours of discovery or notification of the event. The PC-CTU will acknowledge receipt of the SAE Report Form using the PC-CTU ‘SAE Form Receipt’ document. This receipt will be emailed and faxed to the site PI. If the site PI does not receive a receipt within 24hrs of them sending the report (during office hours), they should re-send the SAE Report Form to the PC-CTU by email or fax and telephone ahead.

The documentation will be reviewed by the Quality Assurance Manager (or nominated person) and the ‘SAE Checklist’ will be completed and retained by the PC-CTU. Following the initial check of the report, any additional information will be requested, and the CI or their medically qualified designated representative will review and evaluate the report for seriousness, causality and expectedness, within three additional working days. In the event of a SUSAR the reporting timelines stated below should be followed. If there have been two assessments of causality made, the site PI’s assessment cannot be downgraded. Where there is a discrepancy the worst case assessment is used for reporting purposes.

The PC-CTU will also ensure that SAE reports are reviewed by the TSC at least twice during the study at teleconference meetings. TSC meetings take place at least annually and more often than that as required.

### *SUSARs*

#### All SUSARs will be reported by the CI to the relevant Competent Authority (MHRA) and to the Research Ethics Committee (REC), and other parties as applicable. For fatal and life-threatening SUSARS, this will be done no later than seven calendar days after the Sponsor (or delegate) is first aware of the reaction. Any additional relevant information will be reported within eight calendar days of the initial report. All other SUSARs will be reported within 15 calendar days

## Development safety update reports

The CI will submit (in addition to the expedited reporting above) development safety update reports (DSURs) once a year throughout the clinical trial, or on request, to the Competent Authority (MHRA), REC, Host NHS Trust and Sponsor.

## References

1. Fagerstrom KO, Hughes JR: **Nicotine concentrations with concurrent use of cigarettes and nicotine replacement: a review**. *Nicotine Tob Res* 2002, **4 Suppl 2:**S73-S79.
2. Moore D, Aveyard P, Connock M, Wang D, Fry-Smith A, Barton PM: **Nicotine replacement therapy assisted reduction to stop smoking: A systematic review and meta-analysis of effectiveness and safety.** *BMJ* 2009, **338:**b1024.
3. Zevin S, Jacob III P, Benowitz NL: **Dose-related cardiovascular and endocrine effects of transdermal nicotine**. *Clin Pharmacol Ther* 1998, **64:**87-95.
4. Bullen C, Howe C, Bin Lin R, Grigg M, Laugesen M, McRobbie H, Glover H, Walker N, Wallace Bell N, Whittaker R, Rodgers A: **Pre-cessation nicotine replacement therapy: pragmatic randomized trial.** *Addiction* 2010, **105:1**474-1483.
5. Rose JE, Behm FM, Westman EC: **Nicotine mecamylamine treatment for smoking cessation: the role of pre-cessation therapy**. *ExpClin Psychopharm* 1998, **6:**331-343.
6. Rose JE, Herskovic JE, Behm FM, Westman EC: **Precessation treatment with nicotine patch significantly increases abstinence rates relative to conventional treatment**. *Nicotine Tob Res* 2009, **11:**1067-1075.
7. Schuurmans MM, Diacon AH, Van Biljon X, Bolliger CT: **Effect of pre-treatment with nicotine patch on withdrawal symptoms and abstinence rates in smokers subsequently quitting with the nicotine patch: a randomized controlled trial**. *Addiction* 2004, **99:**634-640.

Table 1: Definitions used to establish the relationship between AEs and trial medication

| **Relationship** | **Description** |
| --- | --- |
| **Unrelated** | There is no evidence of any causal relationship. |
| **Unlikely** | There is little evidence to suggest a causal relationship (eg. the event did not occur within a reasonable time after administration of the trial medication). However, the influence of other factors may have contributed to the event (eg. the participant’s clinical condition, other concomitant treatments). |
| **Possible** | There is some evidence to suggest a causal relationship (eg. because the event occurs within a reasonable time after administration of the trial medication). However, the influence of other factors may have contributed to the event (eg. the participant’s clinical condition, other concomitant treatments). |
| **Probable** | There is evidence to suggest a causal relationship and the influence of other factors is unlikely. |
| **Definitely** | There is clear evidence to suggest a causal relationship and other possible contributing factors can be ruled out. |
| **Not assessable** | There is insufficient or incomplete evidence to make a clinical judgement of the causal relationship. |
