# Supplementary material for: Nicotine patch preloading for smoking cessation (the preloading trial): study protocol for a randomized controlled trial
Source: Trials. 2014 Jul 22;15:296. doi: 10.1186/1745-6215-15-296 (PMC4223826; doi:10.1186/1745-6215-15-296)
Supplement: Additional file 2 — Observational analyses. Details of secondary investigations into genetics and weight gain. [file 1745-6215-15-296-S2.docx]

# Additional file 2: Observational Analyses

## Rational for measuring weight change

Eighty percent of smokers gain weight when they quit [1, 2], which offsets some of the benefits of quitting.[3-5] Meta-analysis of data from the control arms of smoking cessation trials shows that people who achieve continuous abstinence gain a mean of 4kg at six months and 5kg at 12 months.[6] However, we have only scanty and somewhat contradictory data on predictors of weight gain [7]. Although the mean weight gain is relatively modest at 5kg at one year, the variability is large with a standard deviation of 4kg [6]. This shows that some people gain much more than the mean - about 15% gain more than 10kg in a year. It would be helpful to identify these people before they quit smoking and ensure that they were offered programmes known to prevent some weight gain.[8] It seems likely that past weight gain on quitting may be an important risk factor for weight gain in a current quit attempt. One study suggests this but the data are scant.[9] Other baseline characteristics that have been identified as possible predictors in several prospective cohorts include daily cigarette consumption, tobacco addiction severity, age, gender, socioeconomic status, ethnicity, baseline BMI, heaviest weight to date, alcohol consumption.[7, 10-12] We will therefore use this large smoking cessation study to create a model that might be used in the future to predict who would gain excessive weight, and therefore who might be offered special interventions to prevent this. To do so, we will use only those variables that either are routinely or could routinely be measured in a smoking cessation clinic.

The second issue is that some observational data shows those who relapse to smoking revert to their smoking weight.[1, 13] However, more evidence is required to confirm this. This is important because smokers characteristically make many attempts to stop smoking. If each attempt is associated with an increment in weight gain, then attempts to stop smoking could lead to a very large eventual weight gain. This study is not ideal for assessing weight gain and subsequent loss on relapse, because we have no clinical contacts after quit day until the long-term follow-ups at six and 12 months. However, by assessing weight in all smokers at longer follow-ups, we will be able to see whether there has been weight gain beyond that expected from population norms.[14]

## Rationale for the collection of genetic data

Smoking behaviours, including heaviness of smoking and smoking cessation, are known to be under a degree of genetic influence[15], and elucidating the genetic predictors of smoking behaviours may help to develop new pharmacotherapies for smoking cessation, or identify sub-groups for whom more intensive support may be necessary. For example, we recently reported evidence for a moderating effect of catechol-*O*-methyltransferase (*COMT*) rs4680 genotype on the relative efficacy of nicotine replacement therapy (NRT) transdermal patch compared to placebo.[16] NRT produced relatively greater benefit compared to placebo in producing abstinence in individuals with the *COMT* AA (Met/Met) genotype, compared to those with either the AG (Met/Val) or GG (Val/Val) genotype. We subsequently replicated this association of the A (Met) allele with improved response to NRT in an open-label trial of the NRT transdermal patch.[17] However, future studies will most likely require the use of genome wide association methods to identify novel genes associated with smoking cessation, for which large samples are required. Therefore, the collection of additional genetic data within clinical trials of smoking cessation is necessary to augment existing samples, such as the Patch II and Patch in Practice trial samples which some of the investigators hold.[18]

## Objectives

1. To measure weight change over the course of the study, predictors of this weight change, and the impact that smoking relapse has on this.
2. To investigate genetic markers of the success of smoking cessation attempts.

## Assessment

It would be ideal to weigh everyone at baseline and throughout treatment to see how weight changes in relation to abstinence and relapsing. However, no scheduled in-person follow ups are due during the period when most relapses will take place. For abstinent smokers, it will be possible to get accurate weights at baseline and six and 12 month follow-ups using validated weighing scales. For non-abstinent smokers, we will have to rely on telephone follow-up and get a self-reported weight at 6 and 12 months. We plan to ask participants at these follow ups to stand on their own scales during the telephone call or to report last weight, both of which are likely to be party to a margin of error. To compensate for this error, we will ask participants to weigh themselves at home prior to attending clinic at baseline and this self-report weight will be recorded along with their calibrated measured weight (recorded in clinic). The difference between these will be used as a correction to the weights obtained at follow-up. By asking attendees to provide self-reported weight as well as measured weight at follow-up we will be able to calculate the difference again and measure the reliability of the correction. In addition to the measures above, at baseline we shall also measure other potential predictors of weight change. We shall measure height using a height measure. Alcohol intake shall be assessed by asking participants about their alcohol intake over a typical week, including the number of alcoholic drinks per week, how large these drinks are, and on how many days of the week the participant usually drinks, in order to allow an estimation of average units per week. We will also ask participants to provide their heaviest weight to date, and measure previous quit attempt weight gain by asking participants how long they were abstinent for at their most successful (longest) quit attempt, and how much weight they gained during this attempt. We have prepared centile charts of weight gain and the centile chart z-score will be used as the predictor in the modelling. This effectively adjusts for length of abstinence.

## A blood sample will be taken at baseline to identify genetic information . This will only take place at research centres with the resources to do so, where researchers are trained in phlebotomy, and will be optional for participants.

## Analysis

Our first question on weight is whether smokers who become abstinent and gain weight lose it again at follow up. As most people who relapse will do this prior to six month follow up, we will have no contemporaneously collected data on weight attained and therefore cannot examine whether weight was lost. Instead we will approach this indirectly by comparing weight change in people who are relapsed at follow up to population weight gain norms. Weight gain depends upon gender, age, and body mass index (BMI), so for each individual we will calculate an expected weight change using data from a meta-analysis of cohort studies.[19] We will then calculate the difference between observed and expected weight gain, and the mean of these differences for abstinent and non-abstinent participants will be calculated, and compared to an expected difference of 0kg using a one-sample t-test, and compared with each other in a two-sample t-test. This analysis will show whether smokers who become abstinent gain more weight than expected, but this is well established. Less established is what happens to smokers who gain weight but relapse. If their weight gain is greater than expected from population norms, this might suggest that unsuccessful attempts to stop smoking are associated with incremental weight gain, which would imply that abstinent smokers who relapse do not lose the weight that they gained during the quit attempt. We will explore this in two further analyses.

First, we aim to calculate the expected weight gain for relapsed smokers assuming that they do not lose weight on relapse. We can calculate the expected weight gain for each month of abstinence from a meta-analysis of cohort studies of weight gain after abstinence[20]. We will add this to the expected weight gain when not abstinent from a meta-analysis of cohort studies of the general population[19]. If weight is not lost, then the mean observed and expected weight gain will be similar and we will examine this using a one-sample t-test. We will also use a regression equation to examine weight change in relation to length of abstinence, adjusting for age, gender, and baseline BMI. We will use length of abstinence, categorised by dummy variables for months of abstinence achieved, to allow for a non-linear relation.

The second aim relating to weight gain is to create a predictive model that could be used to differentiate people who are at low risk from those who are at higher risk of weight gain if they become abstinent (to be able to offer the latter special interventions). To do so we will use data collected routinely in smoking cessation clinics plus alcohol consumption, which is not routinely collected in these settings but is in primary care. The modelling will use measured weight in abstinent smokers only, assessed at six and 12 months, with separate models for each. Excess weight will be defined as more than four kilograms at six months and five kilograms at 12 months, which is about the median weight gain at these times. We will investigate the effects of varying the threshold. Using logistic regression with backwards elimination (p<0.05), we will investigate the predictors of weight gain using baseline cigarette consumption, FTND (noting the potential for collinearity), age, gender, baseline BMI, socio-economic status, educational qualification, ethnicity and alcohol consumption. We will construct a receiver operating characteristic (ROC) curve to assess the ability of the model to differentiate people at higher risk from those at lower risk of weight gain, and assess the optimum cut-points for these. The data would need confirmation in a separate replication sample, but could easily be incorporated into a simple tool akin to the Framingham score.

Genetic data taken from the blood samples taken will be added to wider samples of such data and genome wide association methods used to identify novel genes associated with smoking cessation.

## References

1. Lycett D, Munafo M, Johnstone E, Murphy M, Aveyard P: **Associations between weight change over 8 years and baseline body mass index in a cohort of continuing and quitting smokers**. *Addiction* 2011, **106:**188-196.
2. US Department of Health and Human Services. **The Health Benefits of Smoking Cessation: a report of the Surgeon General**. *DHHS Publication No (CDC) 90-8416* 1990. Office on Smoking and Health: Rockville , Maryland
3. Davey Smith G, Bracha Y, Svendsen KH, Neaton JD, Haffner SM, Kuller LH, for the Multiple Risk Factor Intervention Trial Research Group: **Incidence of Type 2 Diabetes in the Randomized Multiple Risk Factor Intervention Trial**. *Ann Intern Med* 2005, **142:**313-322.
4. Gerace TA, Hollis J, Ockene JK, Svendsen K: **Smoking cessation and change in diastolic blood pressure, body weight, and plasma lipids**. *Prev Med* 1991, **20:**602-620.
5. Chinn S, Jarvis D, Melotti R, Luczynska C, Ackermann-Liebrich U, Antό JM, Cerveri I, de Marco R, Gislason T, Heinrich J, Janson C, Künzli N, Leynaert B, Neukirch F, Schouten J, Sunyer J, Svanes C, Vermeire P, Wjst M, Burney P: **Smoking cessation, lung function, and weight gain: a follow-up study**.  *Lancet* 2007, **365:**1629-1635.
6. Aubin HJ, Parsons AC, Lycett D, Lahmek P, Aveyard P: **Weight gain in smokers after quitting cigarettes: meta-analysis.**  *BMJ* 2012, **345:** e4439 .
7. Froom P, Melamed S, Benbassat J: **Smoking cessation and weight gain**. *J Fam Pract* 1998, **46:**460-464.
8. Farley AC, Hajek P, Lycett D, Aveyard P: **Interventions for preventing weight gain after smoking cessation**. *Cochrane Database of Systematic Reviews* 2012, Issue 1. Art. No.: CD006219. DOI: 10.1002/14651858.CD006219.pub3.
9. Hall SM, Ginsberg D, Jones RT: **Smoking cessation and weight gain**. *J Consult Clin Psych* 1986, **54:**342-346.
10. Klesges RC, Meyers AW, Klesges LM, La Vasque ME: **Smoking, body weight, and their effects on smoking behavior: a comprehensive review of the literature**. *Psychol Bull* 1989, **106:**204-230.
11. Pistelli F, Aquilini F, Carrozzi L: **Weight gain after smoking cessation**. *Monaldi Arch Chest Dis* 2009, **71:**81-87.
12. Lycett D, Munafo M, Johnstone E, Murphy M, Aveyard P: **Weight change over eight Yyears in relation to alcohol consumption in a cohort of continuing smokers and quitters**. *Nicotine Tob Res* 2011, **13**:1149-1154.
13. O'Hara P, Connett JE, Lee WW, Nides M, Murray R, Wise R: **Early and late weight gain following smoking cessation in the Lung Health Study**. *Am J Epidemiol* 1998, **148:**821-830.
14. Rosell M, Appleby P, Spencer E, Key T: **Weight gain over 5 years in 21,966 meat-eating, fish-eating, vegetarian, and vegan men and women in EPIC-Oxford**. *Int J Obes* 2006, **30:**1389-1396.
15. Munafo MR, Clark TG, Johnstone EC, Murphy MFG, Walton RT: **The genetic basis for smoking behavior: A systematic review and meta-analysis**. *Nicotine Tob Res* 2004, **6:**583-597.
16. Johnstone EC, Elliot KM, David SP, Murphy MFG, Walton RT, Munafo MR: **Association of COMT Val108/158Met genotype with smoking cessation in a nicotine replacement therapy randomized trial**. *Cancer Epidem Biomar* 2007, **16:**1065-1069.
17. Munafo MR, Johnstone EC, Guo B, Murphy MFG, Aveyard P: **Association of COMT Val108/158Met genotype with smoking cessation**. *Pharmacogenet Genom* 2008, **18:** 121-128.
18. David SP, Johnstone EC, Churchman M, Aveyard P, Murphy MFG, Munafo MR: **Pharmacogenetics of smoking cessation in general practice: results from the patch II and patch in practice trials**. *Nicotine Tob Res* 2011.
19. Prospective Studies Collaboration, Whitlock G, Lewington S, Sherliker P, Clarke R, Emberson J, Halsey J, Qizilbash N, Collinns R, Peto R: **Body-mass index and cause-specific mortality in 900 000 adults: collaborative analyses of 57 prospective studies**. *Lancet* 2009, **373**:1083-1096.
20. Aubin HJ, Parsons AC, Lycett D, Lahmek P, Aveyard P: **Weight gain in smokers after quitting cigarettes: meta-analysis.**  *BMJ* 2012, **345:** e4439**.**
